# Retrospective identification of the first cord blood–transplanted severe aplastic anemia in a *STAT1*-associated chronic mucocutaneous candidiasis family: case report, review of literature and pathophysiologic background

**DOI:** 10.3389/fimmu.2024.1430938

**Published:** 2024-07-24

**Authors:** Franz-Martin Fink, Reinhard Höpfl, Martina Witsch-Baumgartner, Gabriele Kropshofer, Sabine Martin, Valentin Fink, Maximilian Heeg, Christina Peters, Johannes Zschocke, Oskar A. Haas

**Affiliations:** ^1^ Department of Pediatrics, Regional Hospital, St. Johann in Tirol, Austria; ^2^ Department of Dermatology and Venerology, Medical University Hospital, Innsbruck, Austria; ^3^ Institute of Human Genetics, Medical University, Innsbruck, Austria; ^4^ Department of Pediatrics, Medical University Hospital, Innsbruck, Austria; ^5^ Institute for Immunodeficiency, Center for Chronic Immunodeficiency, Medical Center-University of Freiburg, Faculty of Medicine, University of Freiburg, Freiburg, Germany; ^6^ Stem Cell Transplantation Unit, St. Anna Children’s Hospital, Department of Pediatrics and Adolescent Medicine, Medical University of Vienna, Vienna, Austria; ^7^ St. Anna Children’s Cancer Research Institute (CCRI), Vienna, Austria; ^8^ Central Laboratory, St. Anna Children’s Hospital, Department of Pediatrics and Adolescent Medicine, Medical University of Vienna, Vienna, Austria; ^9^ Ihr Labor, Medical Diagnostic Laboratories, Vienna, Austria

**Keywords:** chronic mucocutaneous candidiasis (CMC), severe aplastic anemia (SAA), STAT1, gain-of-function pathogenic variant, transplantation

## Abstract

Severe aplastic anemia (SAA) is a life-threatening bone marrow failure syndrome whose development can be triggered by environmental, autoimmune, and/or genetic factors. The latter comprises germ line pathogenic variants in genes that bring about habitually predisposing syndromes as well as immune deficiencies that do so only occasionally. One of these disorders is the autosomal dominant form of chronic mucocutaneous candidiasis (CMC), which is defined by germ line *STAT1* gain-of-function (GOF) pathogenic variants. The resultant overexpression and constitutive activation of STAT1 dysregulate the Janus kinase/signal transducer and activator of transcription 1 (STAT) signaling pathway, which normally organizes the development and proper interaction of different components of the immunologic and hematopoietic system. Although SAA is an extremely rare complication in this disorder, it gained a more widespread interest when it became clear that the underlying causative pathomechanism may, in a similar fashion, also be instrumental in at least some of the idiopathic SAA cases. Based on these premises, we present herein what is the historically most likely first cord blood–transplanted SAA case in a CMC family with a documented *STAT1* GOF pathogenic variant. In addition, we recapitulate the characteristics of the six CMC SAA cases that have been reported so far and discuss the significance of *STAT1* GOF pathogenic variants and other STAT1 signaling derangements in the context of these specific types of bone marrow failure syndromes. Because a constitutively activated STAT1 signaling, be it driven by *STAT1* GOF germ line pathogenic variants or any other pathogenic variant-independent events, is apparently important for initiating and maintaining the SAA disease process, we propose to acknowledge that SAA is one of the definite disease manifestations in *STAT1*-mutated CMC cases. For the same reason, we deem it necessary to also incorporate molecular and functional analyses of *STAT1* into the diagnostic work-up of SAA cases.

## Introduction

Severe aplastic anemia (SAA) is a life-threatening bone marrow failure (BMF) syndrome that can be caused by a radiation-evoked damage of the hematopoietic system, an autoimmune- or infection-associated destruction of bone marrow cells either by hepatitis B, parvo-, cytomegalo-, or Epstein–Barr viruses or by other yet unknown causes (1). There are three genetic disorders that typically predispose affected children to the development of SAA, namely, the Fanconi anemia DNA repair and dyskeratosis congenita telomere maintenance systems and the Shwachman–Diamond syndrome, a disorder of defective ribosome biogenesis (2–5). Moreover, SAA may occasionally also develop in the context of four specific immune disorders. These include the adenosine deaminase 2 deficiency (due to homozygous or compound heterozygous variants in the *ADA2*/*CERC* gene) (6–8), the loss of function (LOF) of the cytotoxic T-lymphocyte–associated protein 4 (caused by autosomal recessive variants in the *CTLA4* gene) (4, 9), the gain of function (GOF) of the Toll-like receptor 8 (10–12), and the signal transducer and activator of transcription 1 (STAT1), which all result from dominant heterozygous gene alterations (13–16).

Recurrent and persistent infections with various candida species are the hallmark of several genetically distinct immunodeficiency disorders (17–24). The autosomal dominant form of chronic mucocutaneous candidiasis (CMC) results from *STAT1* GOF pathogenic variants (OMIM #614162 and orphan designation ORPHA1334) (18, 19, 22, 24–26). They designate neither a genetically nor clinically distinct entity but rather a form of combined immunodeficiency with recurrent or persistent infections of the nails, skin, and mucous membranes that are caused by various candida species and commonly accompanied by diverse other bacterial and viral infections as well as a smorgasbord of autoimmune and inflammatory disorders, cytopenias, aneurysms, and squamous cell carcinomas (19, 22, 26, 27). The most frequent autoimmune manifestations comprise thyroiditis and destructive blood cytopenias, such as autoimmune hemolytic anemia (AIHA), immune thrombocytopenia (ITP), and neutropenia. The few cases of SAA in carriers with *STAT1* GOF variants gained a more widespread interest when it became clear that also pathogenic variant-independent modes of STAT1 activation can trigger the development of idiopathic SAA forms, an insight that is, nowadays, already exploited therapeutically with drugs that inhibit the Janus kinase (JAK) (13–16, 28–31).

We report herein a CMC family with three *STAT1* pathogenic variant carriers, two brothers and their father, who had been transplanted as a child because of a SAA.

## Family history

**Figure 1:**
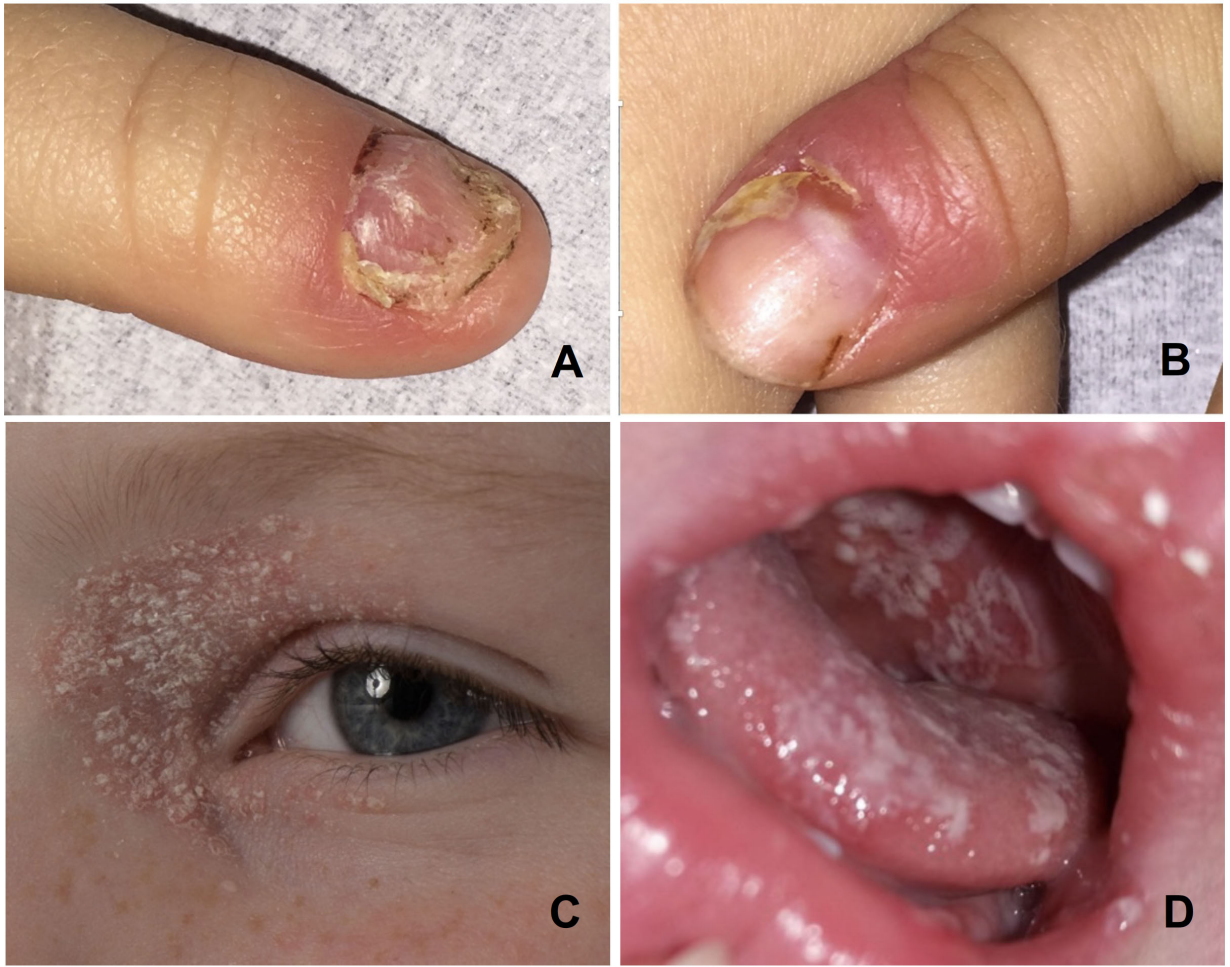
Photographs of the CMC manifestations in the two boys: Candida paronychias and onychodystrophic thumbnails **(A, B)** and periocular seborrheic eczema **(C)** in the index patient 1, and oral candidiasis in his brother **(D)**.

A 4-year-old boy (patient 1) presented with CMC-associated recurring oral candidiasis and candida-associated paronychia since infancy, body-wide scaly seborrheic eczemas that extended to the eyelids, and a dystrophic growth of both thumbnails due to a chronic inflammation of the nail folds (**Figure 1**). His 14-month-old brother (patient 2) developed a severe oral thrush 3 months later (**Figure 1D**). In 1991, 28 years earlier, their father (patient 3) developed a SAA when he was 4 years old. At that time, his white blood cell count was 3.6 × 10^9^/L (age-adjusted normal range, 4.1 to 14.6 × 10^9^/L) with an absolute neutrophil count of 324 × 10^6^/L (normal, 200 × 10^6^/L) and a red blood cell count of 1.08 × 10^12^/L (normal, 3.98 to 5.33 × 10^9^/L). His hemoglobin was 3.6 g/dL (normal, 10.7 g/dL to 14.2 g/dL) and his platelets were 10 × 10^9^/L (normal, 168 to 453 × 10^9^/L) (32). Eleven months earlier, he had a hepatitis A infection; 6 months earlier, a bronchitis; and 2 months, earlier a whooping cough. When he was 3 years old, he had developed a mild psoriasis-like seborrheic eczema but none of the more specific CMC signs. The patient became transfusion-dependent 2 months after first presentation and was, therefore, treated unsuccessfully with high-dose corticosteroids and anti-lymphocyte globulin. Six months later, he was transplanted with cord blood cells from his newly born brother. The conditioning consisted of cyclophosphamide at 200 mg/kg and 5 Gy of total nodal irradiation and the graft-versus-host prophylaxis of ciclosporin A and methotrexate. Stem cell engraftment was delayed, but, following G-CSF treatment, his white blood cell count rose over 1.0 × 10^3^/µL on day +39. He received his last platelet transfusion on day +40 and the last unit of packed red blood cells on day +47. Since then, the now 37-year-old father has normal blood cell counts, had never experienced any CMC symptoms, and remained healthy. Also, the clinical course of the two affected boys is so far benign. They require only occasionally symptomatic antifungal treatment. Fluorescence activated cell sorting (FACS) analyses of peripheral blood mononuclear cells of the two brothers and their transplanted father revealed a normal distribution of lymphocyte subpopulations. Their levels of immunoglobulins and immunoglobulin G (IgG) subclasses were normal and none of them had developed autoantibodies or endocrine deficiencies.

## Genetic analyses

**Figure 2:**
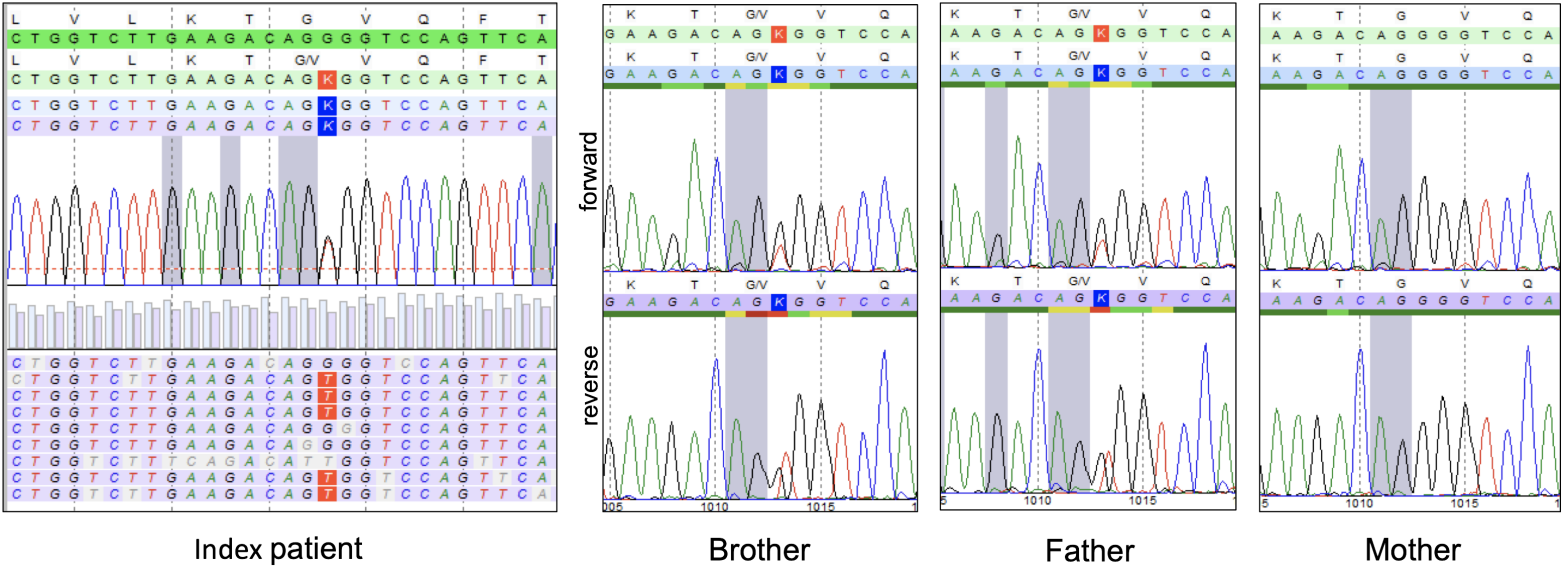
Result of the pathogenic variant screening in our CMC family. In the index patient, targeted pathogenic variant screening revealed a heterozygous c.1013G>T (p.Gly338Val) missense pathogenic variant in the *STAT1* gene, which was subsequently also identified in the peripheral blood of his brother and in the fibroblasts of his father with Sanger sequencing the respective PCR-amplified Exon 11, but not in the healthy mother.

To identify the genetic cause of this familial disorder, we used the Illumina’s TruSight™ One Panel to sequence DNA that was extracted from the older boy’s peripheral blood cells and focused our screening efforts on the three most likely responsible genes, namely, *STAT1*, *IL17RA*, and *AIRE.* This approach uncovered a hitherto undescribed heterozygous *STAT1* missense variant c.1013G>T (p.Gly338Val) that affects the DNA-binding domain (**Figure 2**) (19). Based on the criteria put forward by the American College of Medical Genetics and Genomics (ACMG) standards and guidelines for the interpretation of sequence variants, we initially classified it as variant of unknown significance (33). Twenty-eight years after the father had been transplanted, we succeeded to identify this variant in his skin fibroblasts with an allele frequency of 50%. Neither the children’s parental grandparents nor their mother carried the pathogenic variant. The fact that we found the same variant in both diseased brothers and their father enabled us to reclassify it as likely pathogenic.

### Functional evaluation of the *STAT1* pathogenic variant

**Figure 3:**
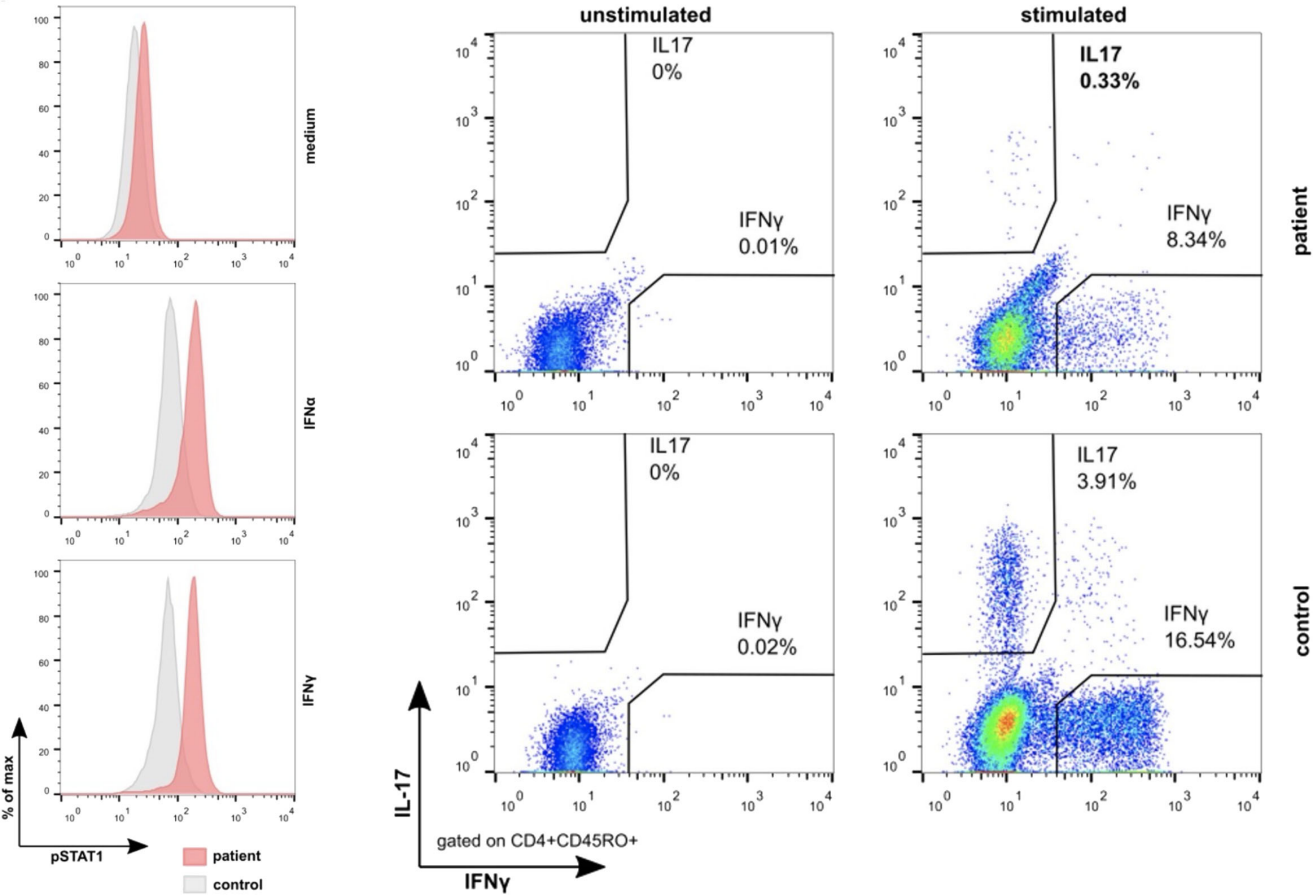
Results of the analyses of the functional consequences of the *STAT1* c.1013G>T (p.Gly338Val) missense pathogenic variant. Compared to healthy controls, stimulation with interferon-α as well as interferon-γ leads to the hyperphosphorylation of STAT1 in the monocytes (left) of the index patient and his brother (not shown). Conversely, stimulation with the NF-κB activator phorbol myristate acetate and ionomycin revealed the impaired expression of interleukin-17 as well as interferon-γ in their CD45RO+CD4+ T cells. Together, these findings provide persuasive evidence for the GOF effect of this pathogenic variant.

To further verify the functional relevance of this novel *STAT1* missense variant, we stimulated peripheral blood mononuclear cells (PB-MNCs) of the two brothers with interferon (IFN)–α and IFN-γ, which led to a significant increase in the phosphorylation of STAT1 (**Figure 3**). Conversely, incubating PB-MNCs with the “nuclear factor ‘kappa-light-chain enhancer’ of activated B cells” (NF-κB) activators phorbol myristate acetate and ionomycin disclosed an impaired expression of interleukin-17 (IL-17) as well as IFN-γ in the patient’s CD45RO+CD4+ T cells (**Figure 3**). Together, these results clearly proved the GOF nature of this c.1013G>T *STAT1* missense variant.

## Discussion

The most remarkable finding in the CMC family reported herein is that the father was a *STAT1* GOF variant carrier (case 7, **Table 1**). As a child, he was transplanted with his brother’s cord blood cells because of a presumably infection- or autoimmune-induced SAA. If it had not been for his diseased sons, then we would have never contemplated that he might be a *STAT1* GOF variant carrier. Yet, 28 years later, we were able to secure the GOF variant in his skin fibroblasts and, by that, identify the historically first SAA case with a *STAT1* GOF variant who had received a life-saving cord blood transplant.

**Table 1:** Overview of seven cases with CMC, GOF-STAT1 mutations and SAA.

Case	Sex	Age (years)	Clinical picture	*STAT1 Pathogenic variant*	Location (domain)	Treatment	Reference
1	Male	56	CMC, pernicious anemia, thymoma, SAA	not available	-	Plasmapheresis	(34)
2	Female	7	CMC, SAA	not available	–	Bone marrow transplantation (1982)	(35)
3	Female	10	CMC, AIHA, ITP, suspected MAS, SAA	c.1633G>A (p.Glu545Lys)	Linker	ATG + CyA, JAK1/2-inhibitor (ruxolitinib)	(16)
4	Male	18	CMC, SAA	c.800C>/T (Ala267Val)	Coiled coil	JAK1-inhibitor (icatinib)	(15)
5	Female	8	CMC, SLE, AIHA, hypothyroidism, PRCA	c.854A>G (p.Q285R)	Coiled coil	JAK1/2-inhibitor (ruxolitinib), stem cell transplantation	(14)
6	Female	32	no CMC, oral ulcers, Typ-1 diabetes, SAA	c.520T>C (p.Cys174Ala)	Coiled coil	Not reported	(13)
7	Male	4	no CMC, SAA	c.1013G>T (p.Gly338Val)	DNA binding	Cord blood transplantation (1991)	Present report (father)

AIHA, autoimmune hemolytic anemia; ITP, immune thrombocytopenia; MAS, macrophage activating syndrome; SLE, systemic lupus erythematosus; ATG, anti-thymocyte globulin; CyA, ciclosporin A.

In individuals with CMC, genuine BMF syndromes, such as SAA and pure red cell aplasia (PRCA), seem either extremely rare or underreported (19, 22, 26, 36). There are only two such cases that were published earlier. The first one was reported in 1975 by Twomey et al. (case 1, **Table 1**) (34). An already 56-year-old man developed SAA and a small thymoma 9 years after mucocutaneous candidiasis was first noted. The second case concerned a 7-year-old girl with CMC and SAA, who was successfully treated with a bone marrow transplant (case 2, **Table 1**) (35).

In addition to these two cases and the one presented herein, only three other *STAT1*-associated CMC cases with SAA and one with a PRCA were reported (**Table 1**) (13–16). The first of these concerns a 10-year-old girl who originally suffered from a life-threatening Evans syndrome with episodes of AIHA and ITP (case 3, **Table 1**) (16). Later on, she developed a SAA together with a suspected macrophage activation syndrome. She was treated with steroids, intravenous immunoglobulins, the anti-CD20 antibody rituximab, the anti-complement C5 antibody eculizumab, and, thereafter, with anti-thymocyte globulin and ciclosporin A. Only the latter restored her bone marrow function to some extent but did not produce a complete remission. Following the detection of a *STAT1* GOF pathogenic variant and the subsequent meticulous *in vitro* assessment of its functional consequences, she received the JAK1/2 inhibitor ruxolitinib. Continuous treatment with this inhibitor alone resolved all her autoimmune-mediated problems, and she was reported to be still in complete remission 18 months later.

The second case concerns an 18-year-old man with oral ulcers and CMC who developed SAA with an only moderately decreased number of platelets, but weekly transfusion-dependent anemia and several episodes of febrile neutropenia (case 4, **Table 1**) (15). He was treated with the JAK1 inhibitor itacitinib for 20 months without any adverse effects that resulted in a rapid and remarkable recovery of hematopoiesis, before he self-discontinued this treatment. Three years after initial presentation and approximately 12 months off itacitinib therapy, he was still in continuous hematologic remission. Based on the discovery and insight that a significant proportion of other patients with “idiopathic” forms of SAA share similar STAT1-mediated pathophysiologic changes, the authors were the first to suggest that such “idiopathic” cases might also benefit from treatment with JAK inhibitors in a similar manner.

The third case concerns a 32-year-old woman with oral ulcers, oral candidiasis, recurrent pneumonia, type 1 diabetes mellitus, and SAA with a relatively preserved thrombocyte level (case 5, **Table 1**) (13). The authors did not provide any further clinical information, but they also found that STAT1 can be overexpressed in patients with idiopathic SAA.

Because PRCA and SAA are BMF syndromes with a closely related etiology, we also include the girl with CMC and a PRCA (case 6, **Table 1**) (14). With 1 year, she already suffered from a systemic lupus erythematosus before she developed CMC-typical infections and eventually PRCA with 8 years. Because conventional immunosuppressive therapies were not effective, she received the JAK inhibitor ruxolitinib and was transplanted after she had transiently improved but succumbed to transplant-related complications.

*STAT1* encodes one of the seven STAT family transcription factors. They are downstream components of the JAK signaling pathway, which regulates the expression of more than 50 cytokines, growth factors and IFNs (37). After binding to their cognate receptors, the respective ligands dimerize and activate JAKs, which phosphorylate the STAT proteins and, by that, turn on their individual transcriptional programs (15, 37, 38). An activated STAT1 not only impacts the expression of other transcription factors but also self-regulates its own expression (39).

The constantly enhanced STAT1 signaling in *STAT1* GOF variant carriers creates two closely interlinked feedback loops that enhance the IFN-controlled and inhibit the IL-17–controlled immune defense (25, 40–47). Within this context, STAT1 acts both as a signal transducer and transcription activator for the IFN type I (IFN-α and IFN- β), type II (IFN-γ), and type III (IFN-λ) as well as for IL-27 (25, 40, 41, 48). The increased levels of these factors eventually cause all the varied inflammatory, autoimmune, and tissue destructive ailments that CMC patients have to endure (19, 22, 40, 41). The fact that STAT1 signaling occurs through both the type I and the type II IFN receptors prompted Largent et al. to examine the differences between these two transmission routes (43). They found that the deletion of the type I IFN receptors in mice with a *Stat1* GOF pathogenic variant only partly protected them from STAT1-driven systemic inflammations, whereas deletion of the type II IFN receptors normalized total STAT1 expression, which safeguarded them from autoimmune manifestations (43). These findings suggest that IFN-γ is the relevant driver that upregulates STAT1 in a *STAT1* GOF environment (41, 43). The long-term exposure of hematopoietic stem cells to high IFN-γ levels inhibits their self-renewal capacity and pushes them toward terminal differentiation (49–52). It is, therefore, conceivable that an exhaustion of the stem cell compartment is one of the reasons why, in some *STAT1* GOF variant carriers, the bone marrow will fail. We presume that, in our SAA case, this process was most likely instigated and reinforced by the three infections that he had gone through beforehand.

Whether and to which extent STAT1 activity is primarily governed alone by an enhanced production, by the subsequent phosphorylation, and/or by an impaired dephosphorylation of the respective proteins is still a controversial and unresolved issue. While an *in vitro* IFN-α and IFN-γ stimulation–associated phosphorylation is a diagnostic hallmark of every *STAT1* GOF variant (46), Scott et al. and others suggested that STAT1 increased phosphorylation might be a secondary event and only take place when the STAT1 level is already elevated. They, therefore, consider the overall amount of unphosphorylated STAT1 as probably being more relevant for the primary pathogenic effects than phosphorylation itself (39, 53, 54).

Recent *in vitro* experiments provided some evidence that specific *STAT1* GOF variants may predetermine specific disease patterns and clinical phenotypes (25, 39, 55, 56). This notion derives from the variant-specific IFN-α and IFN-γ gene expression patterns that were caused by two different *STAT1* GOF variants in a STAT1-deficient U3A fibrosarcoma cell line transfection model (56). These observations were later extended by Scott et al., who generated five heterozygous CRISPR/Cas9 base–edited *STAT1* GOF variants in diploid HAP1 cells and found that baseline as well as IFN-α or IFN-γ induced expression levels of five investigated IFN-stimulated genes varied starkly depending on the specific pathogenic variant (39). Taken together, these widely divergent variant-specific transcriptional responses suffice at least to comprehend why the resultant disease spectra are so diverse and why it is virtually impossible to predict the overall clinical course in *STAT1* GOF–mutated individuals.

The second feedback loop revolves around the highly versatile context- and tissue-dependent function of the proinflammatory cytokine IL-17 (42, 47, 57). IL-17 is primarily protective against fungal as well as, to a lesser extent, also other types of mucocutaneous infections, which is the reason why individuals with an insufficient IL-17 defense develop such otherwise uncommon diseases (21, 42, 46, 57, 58). However, even in cases with an intact IL-17 response, the local effects of an IFN-γ/STAT1–driven interferonopathy alone can be enough to promote mucocutaneous fungal infections by impairing the integrity of the epithelial barrier (42, 57, 59). Because, in *STAT1* GOF–mutated individuals, both these immune signaling alterations usually concur, their effects may jointly contribute to their heightened susceptibility and the severity of fungal infections.

A hyperactive STAT1 downregulates the IL-17 signaling pathway by interfering with the STAT3-mediated differentiation program of Th17 cells, as already evidenced by the similar immunological and clinical effects of *STAT3* LOF and *STAT1* GOF variants (41, 46, 60–62). Activation of STAT1 and STAT3 by IL-6, IL-21, IL-27, and other factors affect the differentiation program of Th17 cells in opposing ways that range from strong induction (IL-6) to strong inhibition (IL-27) (21, 47, 61, 63, 64). This tightly STAT1- and STAT3-regulated cellular differentiation and interaction network relies on a well-balanced and appropriately adjusted intracellular ratio of the STAT1 and STAT3 transcription factors. Because STAT1 and STAT3 form homo- as well as heterodimers, an overabundance of STAT1 will upregulate the STAT1 transcription program either in a direct or indirect way, for instance, by competing with STAT3 for common receptor docking sites on their respective target promotors (41, 62, 65).

Noteworthy, IL-27 priming impairs also Th17 differentiation by upregulating the programmed cell death protein ligand 1 (PD-L1) in CD4-positive T cells (66, 67). Because this occurs in a STAT1-dependent manner, PD-L1 expression is markedly increased in STAT3 LOF and *STAT1* GOF naïve CD4^+^ T cells. Although such Th17 differentiation defects might theoretically be overcome by blocking PD-L1 with checkpoint inhibitors, this is currently not a viable option in auto-immune diseases because of adverse effects of the respective drugs (66, 68, 69).

Patients with CMC with a *STAT1* GOF pathogenic variant are at risk to develop a broad spectrum of infectious and non-infectious complications. The first sign in infants is usually a recurrent or persisting oral candidiasis (thrush) alone or in combination with chronic nail-fold inflammation. As long as the course of CMC is mild and remains controllable, symptomatic treatment with antifungal, antibacterial, antiviral, and perhaps immune-dampening remedies is generally sufficient (17, 70). The only potential curative option for cases with more severe, exacerbating, and life-threatening disease forms was, for a long time, a hematopoietic stem cell transplantation. With a high rate of secondary graft failures and survival rates of only up to 40%, the overall outcome of this procedure is still poor (26, 36, 71). The main arguments that are commonly put forward to explain these disappointing outcome data are the advanced disease stage and consequently also the poorer health state of patients who are selected for transplantation (36, 71). Thus, some of the factors, which assured that the transplantation went smoothly and without complications in our patient, were his young age, his overall good clinical condition, and the lack of any noteworthy additional ailments before he received his cord blood graft.

The identification of *STAT1* pathogenic variants together with their subsequent meticulous functional assessment paved the way for the development for well-defined targeted treatment approaches (17). The currently best explored ones are JAK inhibitors, such as the JAK1/2 inhibitor ruxolitinib. It downregulates JAK signaling and thereby also reduces STAT1 activity (14–16, 31, 72–78). Hence, JAK inhibitors help to manage the disease by suppressing autoimmune processes and by improving host defense mechanisms. Although sufficient data on the risk-benefit ratio, especially on the long-term application are still pending, JAK inhibitors are already increasingly used in experienced treatment centers in an off-label setting for bridging or as an alternative for stem cell transplantation (72, 73, 77). Another potentially interesting drug that could become helpful in the pretransplant preparation of patients with STAT1 GOF pathogenic variants is the IFN-γ blocker emapalumab (79).

Taken together, we presented herein what is the historically first cord blood–transplanted SAA case in a CMC family with a *STAT1* GOF pathogenic variant. We reviewed the characteristics of all available six cases with CMC and a BMF and briefly discussed the role of *STAT1* GOF pathogenic variants and other STAT1 signaling derangements that are encountered in pathogenic variant-driven and sporadic SAA cases, respectively. Based on this information, we deem it necessary to acknowledge that SAA is one of the definitive hematologic manifestations of *STAT1* GOF–associated CMC and to perform molecular and functional analyses of *STAT1* in all unclear cases of BMFs in the future.

## Data availability statement

The original contributions presented in the study are included in the article/supplementary materials. Further inquiries can be directed to the corresponding authors.

## Ethics statement

Ethical approval was not required for the study involving human samples in accordance with the local legislation and institutional requirements because all the molecular-genetic analyses, functional studies and therapeutic interventions were part of the advanced standard care of the involved patients. Written informed consent for participation in this study was provided by the participants’ legal guardians/next of kin. Written informed consent was obtained from the individual(s), and minor(s)’ legal guardian/next of kin, for the publication of any potentially identifiable images or data included in this article. Written informed consent was obtained from the participant/patient(s) for the publication of this case report.

## Author contributions

F-MF: Writing – original draft, Writing – review & editing, Supervision, Project administration, Investigation, Funding acquisition, Formal analysis, Data curation, Conceptualization. RH: Writing – review & editing, Supervision, Investigation, Data curation, Funding acquisition. MW-B: Writing – review & editing, Methodology, Investigation, Formal analysis, Data curation. GK: Writing – review & editing, Data curation. SM: Writing – review & editing, Data curation. VF: Writing – review & editing, Data curation. MH: Writing – review & editing, Methodology, Investigation, Formal analysis, Data curation. CP: Writing – review & editing, Data curation. JZ: Writing – review & editing, Supervision, Resources, Methodology, Investigation, Funding acquisition. OH: Writing – review & editing, Writing – original draft, Supervision, Formal analysis, Data curation, Conceptualization.
